# Uterine Dysfunction in Biglycan and Decorin Deficient Mice Leads to Dystocia during Parturition

**DOI:** 10.1371/journal.pone.0029627

**Published:** 2012-01-13

**Authors:** Zhiping Wu, Abraham W. Aron, Elyse E. Macksoud, Renato V. Iozzo, Chi-Ming Hai, Beatrice E. Lechner

**Affiliations:** 1 Department of Pediatrics, Women and Infants' Hospital of Rhode Island, The Warren Alpert Medical School of Brown University, Providence, Rhode Island, United States of America; 2 Department of Pathology, Anatomy and Cell Biology, Thomas Jefferson University, Philadelphia, Pennsylvania, United States of America; 3 Department of Molecular Pharmacology, Physiology, and Biotechnology, Brown University, Providence, Rhode Island, United States of America; University of Medicine and Dentistry of New Jersey, United States of America

## Abstract

Cesarean birth rates are rising. Uterine dysfunction, the exact mechanism of which is unknown, is a common indication for Cesarean delivery. Biglycan and decorin are two small leucine-rich proteoglycans expressed in the extracellular matrix of reproductive tissues and muscle. Mice deficient in biglycan display a mild muscular dystrophy, and, along with mice deficient in decorin, are models of Ehlers-Danlos Syndrome, a connective tissue anomaly associated with uterine rupture. As a variant of Ehlers-Danlos Syndrome is caused by a genetic mutation resulting in abnormal biglycan and decorin secretion, we hypothesized that biglycan and decorin play a role in uterine function. Thus, we assessed wild-type, biglycan, decorin and double knockout pregnancies for timing of birth and uterine function. Uteri were harvested at embryonic days 12, 15 and 18. Nonpregnant uterine samples of the same genotypes were assessed for tissue failure rate and spontaneous and oxytocin-induced contractility. We discovered that biglycan/decorin mixed double-knockout dams displayed dystocia, were at increased risk of delayed labor onset, and showed increased tissue failure in a predominantly decorin-dependent manner. *In vitro* spontaneous uterine contractile amplitude and oxytocin-induced contractile force were decreased in all biglycan and decorin knockout genotypes compared to wild-type. Notably, we found no significant compensation between biglycan and decorin using quantitative real time PCR or immunohistochemistry. We conclude that the biglycan/decorin mixed double knockout mouse is a model of dystocia and delayed labor onset. Moreover, decorin is necessary for uterine function in a dose-dependent manner, while biglycan exhibits partial compensatory mechanisms *in vivo*. Thus, this model is poised for use as a model for testing novel targets for preventive or therapeutic manipulation of uterine dysfunction.

## Introduction

The rate of birth by Cesarean section has been rising steadily in the United States despite the Healthy People 2020 goal of decreasing Cesarean births. It has increased by 60% since 1996 to a rate of 32% of all births in 2009 [1]. Common indications include previous Cesarean birth, breech positioning, fetal distress and multiple gestation, as well as various clinical scenarios such as failed induction and failure to progress, which are indicators of uterine dysfunction [2].

In the physiologic setting, the uterus is dormant during gestation to avoid preterm birth, then transitions to active contractions when expulsion of the fetus is necessary. These processes are modulated by neuronal, hormonal, metabolic and mechanical factors [3]. A variety of mechanisms have been implicated in the pathogenesis of uterine dysfunction, or dystocia. These include increasing maternal age [4], stress and obesity [5]. In mouse models, genetic mutations may lead to dystocia. Mice with mutations of Atp11c have dystocia [6], while overexpression of small conductance calcium-activated Kþ channel isoform 3 leads to inefficient uterine contractility [7]. Mice with mutations of steroid 5α-reductase, relaxin, cytosolic phospholipase A2 and the prostaglandin F2 α receptor FP display dystocia and/or delayed labor onset (reviewed in [8]). However, the precise mechanism of uterine dysfunction, which is most likely multifactorial, is unclear.

Biglycan is a small leucine-rich proteoglycans (SLRP) that is a component of the extracellular matrix in a variety of tissues including skin, bone, and skeletal as well as cardiac muscle [9], [10], [11]. Its core protein contains two chondroitin or dermatan sulfate side chains [12]. Biglycan binds to collagen VI, transforming growth factor-α (TGF-α), TGF-β, chordin and BMP-4 [13], [14], [15], [16], [17], [18]. Decorin is a small leucine-rich proteoglycan with one chondroitin or dermatan sulfate side chain that demonstrates ∼55% homology with biglycan [19] and also interacts with a number of extracellular matrix constituents and growth factors [20], [21].

Biglycan and decorin are expressed in a variety of gestational tissues in humans, including the placenta [22] and fetal membranes [23], [24], [25], [26]. Biglycan is expressed in the pregnant mouse uterus [27], [28], while both biglycan and decorin are decreased in the human myometrium during labor [29].

Mice deficient in biglycan, decorin, or both, are a model of Ehlers-Danlos syndrome (EDS), a heterogeneous group of rare inherited connective tissue disorders associated with aneurysms as well as uterine rupture and a decrease in tensile strength and integrity of skin, joints, and other connective tissues. These mice display connective tissue anomalies of skin, bone and tendon [30].

In patients afflicted with the progeroid variant of EDS, the molecular basis of the connective tissue anomaly is a mutation of xylosylprotein-4ß-galactosyltransferase I, an enzyme that is necessary for the posttranslational modification leading to the glycosylation of biglycan and decorin. This mutation leads to the abnormal secretion of biglycan and decorin core protein lacking glycosaminoglycan side chains [31], [32].

Women with EDS type IV are at increased risk of uterine rupture [33], [34] and atonic uterus at Cesarean section [35], while infants born with type III Ehlers-Danlos syndrome may be at increased risk of malpresentation during labor [36].

We previously reported that biglycan is developmentally regulated in mouse skeletal muscle as well as in skeletal and diaphragm muscle in human fetuses [37]. Furthermore, the biglycan knockout mouse displays mild muscular dystrophy [38]. In the decorin knockout mouse, the decidualized stroma of the uterus shows abnormal architecture with large diameters and irregular contours of the endometrial collagen fibrils [39]. Furthermore, we have shown that the biglycan/decorin double knockout mouse displays preterm birth [40]. However, little is known about the role that these proteoglycans play in the function of uterine muscle. Thus, we hypothesized that biglycan and decorin could be protective of uterine smooth muscle dysfunction and that the absence of biglycan and decorin would lead to labor dystocia and adverse gestational outcomes. Our findings demonstrate for the first time that the absence of these two key SLRPs favors dystocia. Thus, this double knockout mouse could represent a novel and invaluable tool for testing new therapeutic approaches for the treatment and prevention of human dystocia.

## Materials and Methods

### Ethics Statement

Women and Infants' Hospital Institutional Animal Care and Use Committee approval was obtained and steps were taken to minimize the suffering of animals (Approval # 0077-09).

### Mouse Husbandry

C3H wild-type mice were purchased from Jackson Laboratories (Bar Harbor, ME). A homozygous biglycan knockout (*Bgn−/−Dcn+/+*) breeding pair of C3H background (generated by Marian Young [41]) was a gift from Justin Fallon. A heterozygous decorin knockout (*Bgn+/+Dcn+/−*) breeding pair of C57BL background was mated to the birth of homozygous pups (*Bgn+/+Dcn−/−*), which were then bred to establish the homozygous decorin knockout colony. Mice were housed under standard conditions. A homozygous biglycan knockout female was crossed with a homozygous decorin knockout male to establish breeding pairs in which the females were heterozygous for both biglycan and decorin (*Bgn+/−Dcn+/−*) and the males were heterozygous for decorin but homozygous knockouts for biglycan (*Bgn−/^0^Dcn+/−*), given that biglycan is an X-chromosomal gene. These pairs were mated to breed mixed genotype litters to gain *Bgn−/−Dcn+/−*, *Bgn+/−Dcn−/−* and *Bgn−/−Dcn−/−* females as well as *Bgn−/−Dcn+/+* females of mixed C3H/C57BL background. Wild-type females, *Bgn−/−Dcn+/+* females and *Bgn+/+Dcn−/−* females were bred with males of their own genotype. *Bgn+/−Dcn+/−*, *Bgn+/−Dcn−/−*, *Bgn−/−Dcn+/−* and *Bgn−/−Dcn*−/− females were bred with *Bgn−/^0^Dcn+/−* males given our observation that *Bgn−/^0^ Dcn−/−* males do not produce pregnancies. Breeding pairs were set up in the evening. Plugs were checked the following morning and every morning thereafter. The day of the plug was defined as embryonic day 0. Cages were observed each morning for litters as well as parturition activity. The morning that a litter was observed was defined as the day of birth (P0). Pairs were set up for mating at 5–7 weeks. A subgroup of pregnant dams was sacrificed at E12, E15 and E18. Data was collected on length of pregnancy and presence or absence of dystocia.

### Genotyping

A 3-mm tail biopsy specimen was obtained for each pup within a mixed genotype litter at weaning (around postnatal day 21). Genomic DNA was extracted from each tail biopsy sample using the High Pure PCR Template Preparation Kit (Roche, Mannheim, Germany). Polymerase chain reaction (PCR) was performed to identify the decorin and biglycan alleles using the Taq DNA Polymerase kit (New England Biolabs, Ipswich, MA) and the PTC-200 thermal cycler. The PCR product was run on a 1.8% w/v agarose gel to visualize the following diagnostic bands. The decorin PCR produced bands of 161 bp for the wild-type allele and 238 bp for the knockout allele. The biglycan PCR produced bands of 212 bp and 310 bp for the wild-type and knockout alleles, respectively.

### Tissue Failure Rate to Standard Load and Contraction

8–13 week old virgin female mice of the following genotypes and background strains were used: wild-type (*Bgn+/+Dcn+/+* C3H), *Bgn−/−Dcn+/+* C3H, *Bgn+/+Dcn−/−* C57BL and *Bgn−/−Dcn−/−* C3H/C57BL. In order to elucidate a possible contribution of the background to the observed phenotype, a crossed background *Bgn−/−Dcn+/+* (C3H/C57BL) was also generated. After sacrifice, the uteri were exposed by laparotomy and dissected from the abdominal cavity with care taken to avoid stretching the tissue. The tissue was placed in cold (4°C) physiological salt solution (PSS) containing (in mM): 140 NaCl, 4.7 KCl, 1.2 Na_2_HPO_4_, 2.0 MOPS (pH 7.4), 0.02 Na2EDTA, 1.2 MgSO_4_, 1.6 CaCl_2_, and 5.6 D-glucose. Connective tissue and the cervix were removed under a dissection microscope using microdissecting scissors to expose an individual segment from each uterine horn.

Two stainless steel wire clamps were used to hold each uterine horn segment in place. One wire clamp was connected to a force transducer (Grass FT 03, Grass Technologies, West Warwick, RI) while the other was secured by a glass rod mounted on a length manipulator (Narishige, Tokyo, Japan). The force transducers were connected to a laptop via a Model 7E Poly Graph amplifier with a Polyview Adapter Unit (PVA-16, Grass Technologies, West Warwick, RI). For the studies of tissue failure rate to standard load, the uterine segments were exposed to a standard of 10 g of force. Tissue failure (tearing) or lack thereof was recorded. For the contraction force studies, the same experimental setup was used. After exposure to 10 g of loading force, the tissue was allowed to equilibrate for 1 h in PSS at 37°C and bubbled with air (21% oxygen, 79% nitrogen) to avoid hypoxia. After equilibration, the segments were activated for 3 min by K-PSS (similar in composition to PSS except that 104.95 mM NaCl was substituted for by an equimolar concentration of KCl) [42]. After activation, the tissue was relaxed in PSS for one hour (baseline); then, the uterine segments were exposed to increasing dosages of oxytocin at concentrations of 0.01 nM, 1 nM and 100 nM for 15 min each. After the final oxytocin dose, the tissue was allowed to recover for 30 min in PSS.

### Data Analysis

For the contraction studies, contraction activity was recorded in Polyview Recorder (Grass Technologies, West Warwick, RI). Using Polyview Reviewer (Grass Technologies, West Warwick, RI), the average amplitude of the wave forms was measured in PSS over a 40 minute period (starting at 10 min after the start of the recording). Also, the mean integral of the contraction tracings was calculated over the following intervals: One hour equilibration: 10 min after start to 10 min before K-PSS added. K-PSS: From addition of K-PSS to beginning of relaxation in PSS (baseline). One hour relaxation: 10 min after start to 10 min before addition of 0.01 nM oxytocin. 15 min oxytocin trials: 5 min after start to beginning of next trial. Recovery: 10 min after start to end of recording. Each mean integral was normalized to the trial's mean integral during the one hour equilibration. In cases in which two uterine segments from one mouse were used, their mean integrals were averaged before normalization. Results for *Bgn−/−Dcn+/+* females of both C3H and C3H/C57BL background strains were compared.

### RNA/cDNA Preparation and Quantitative PCR

Wild-type, *Bgn−/−Dcn+/+* and *Bgn+/+Dcn−/−* uterus was dissected at three prenatal time points (E12, E15 and E18) in 0.1 M PBS, pH 7.4, snap-frozen in liquid nitrogen and stored at −80°C. RNA extraction was performed using the Trizol method (Invitrogen, Carlsbad, CA, Carlsbad, CA). Genomic DNA was removed by incubating the RNA sample with DNase I (Invitrogen, Carlsbad, CA) for 30 min at 37°C with subsequent RNA re-extraction with Trizol. The purified RNA was converted to cDNA using the Superscript III First-Strand Synthesis System Kit (Invitrogen, Carlsbad, CA). qPCR reactions were performed on the ABI PRISM 7000 real-time thermocycler (Applied Biosystems, Foster City, CA) and on the Eppendorf Mastercycler epgradient S (Eppendorf, Hamburg, Germany) using the SYBR-Green method (Invitrogen, Carlsbad, CA). Primers were designed using Primer-Blast primer design software (National Library of Medicine, Bethesda, MD). The values were normalized to the housekeeping gene *Gapdh*. Melting point analysis of the product was performed to ensure the absence of alternative products or primer dimers. Data analysis was performed using the comparative Ct method with a validation experiment. qPCR analysis was performed in triplicate. n = 4−6 samples from 4–6 dams per genotype.

### Primer Sequences

qPCR biglycan forward: ATTGCCCTACCCAGAACTTGAC; qPCR biglycan reverse: GCAGAGTATGAACCCTTTCCTG; qPCR decorin forward: TTCCTACTCGGCTGTGAGTC; qPCR decorin reverse: AAGTTGAATGGCAGAACGC; qPCR GAPDH forward: CTCACAATTTCCATCCCAGAC; qPCR GAPDH reverse: TTTTTGGGTGCAGCGAAC; biglycan genotyping PCR wild-type allele forward TGATGAGGAGGCTTCAGGTT; biglycan genotyping PCR wild-type allele reverse GCAGTGTGGTGTCAGGTGAG; biglycan genotyping PCR knockout allele forward TGTGGCTACTCACCTTGCTG; biglycan genotyping PCR knockout allele reverse GCCAGAGGCCACTTGTGTAG; decorin genotyping PCR allele forward CCTTCTGGCACAAGTCTCTTGG; decorin genotyping PCR wild-type allele reverse TCGAAGATGACACTGGCATCGG; decorin genotyping PCR knockout allele reverse TGGATGTGGAATGTGTGCGAG. All primers were provided by Invitrogen (Carlsbad, CA).

### Immunohistochemistry

Wild-type, *Bgn−/−Dcn+/+* and *Bgn+/+Dcn−/−* mouse uteri were dissected at three prenatal time points (E12, E15 and E18) in 0.1 mol l^−1^ phosphate-buffered saline, pH 7.4. The specimens were then flash frozen in isopentane and stored at −80°C. The frozen tissue was cryostat sectioned to 10 µm thickness, mounted on slides and stored at −20°C. Sections were fixed in 1% v/v paraformaldehyde and stained with primary antibodies using the Mouse on Mouse immunostaining kit for monoclonal antibodies (Vector Laboratories, Burlingame, CA), then incubated with primary antibody overnight at 4°C and with secondary antibody for 30 min at room temperature. Sections were mounted with Vectashield mounting medium with DAPI (Vector Laboratories, Burlingame, CA). Fluorescent microscopy to evaluate the samples was performed using an inverted stage Nikon Eclipse TE2000-E microscope equipped with epifluorescent filters and a Nikon Plan Apo 20× and 40× and a Plan Fluor 10× objective lens (Yokohama, Japan). Images were acquired using a Coolsnap HQ cooled CCD camera (Roper Scientific, Ottobrunn, Germany) and MetaVue software (Molecular Devices, Downingtown, PA). Experiments were repeated three times with tissue samples from three dams per genotype. The following antibodies were used: polyclonal rabbit anti-mouse biglycan antibody LF-159 (a gift from Larry Fisher), polyclonal goat anti-mouse decorin antibody (R&D Systems, Minneapolis, MN). Secondary antibody labeling was performed with goat anti-rabbit IgG conjugated to Alexa 488 (Invitrogen, Carlsbad, CA) for biglycan and rabbit anti-goat IgG conjugated to CY3 (Sigma-Aldrich, St. Louis, MO) for decorin. Mouse IgG and rabbit IgG (Vector Laboratories, Burlingame, CA), respectively, were used as controls.

### Western blotting

Wild-type, *Bgn−/−Dcn+/+* and *Bgn+/+Dcn−/−* mouse uteri were dissected at E18 and frozen at −80°C. Tissue samples were cut into small pieces and placed in 1.0 ml T-PER tissue protein extraction buffer (Pierce, Rockford, IL) with one tablet of proteinase inhibitor cocktail per 10 ml buffer (Roche, Basel, Switzerland). Tissue samples were then homogenized for 3×10 s in an ice bath and kept on ice for 30 min. The homogenate was centrifuged at 10,000× *g* at 4°C for 8 min. The supernatant was collected and stored at −80°C until use. Total protein content was determined using the BCA assay (Pierce, Rockford, IL). 30 µg of total protein was loaded on duplicate 10% SDS-polyacrylamide gels and subsequently blotted to a polyvinylidene difluoride membrane. Following blocking in 5% milk in PBS-T buffer (PBS with 0.2% Tween 20) for 30 min, each blot was incubated overnight at 4°C with mouse polyclonal anti-TGF-β1,2,3 antibody (MAB1835, dilution 1∶1000) (R&D Systems, Minneapolis, MN). The blot was then washed with PBS-T buffer briefly and incubated with anti-mouse IgG horseradish peroxidase conjugate (Cell Signaling, Danvers, MA) for 40 min. The Super-Signal West Pico chemiluminescent substrate kit (Pierce, Rockford, IL) was used prior to development of the blot membrane. The bands were compared with protein markers of known molecular size run in parallel on the same SDS-polyacrylamide gel (Bio-Rad, Hercules, CA). The membrane was stripped using Restore Western Blot stripping buffer (Thermo Scientific, Waltham, MA) for 10 min and blocked with 5% milk PBS-T for 20 min, then re-probed with anti-GAPDH antibody at a 1∶800 dilution (Santa Cruz Biotech, Santa Cruz, CA) as an internal standard. Immunoblots were digitally scanned and densitometrically analyzed using Gel-Pro Analyzer (Media Cybernetics, Bethesda, MD). Relative optical density (TGF-β to GAPDH) was normalized to the wild-type band. The experiment was repeated with three individual sets of samples.

## Results

### Abnormal Rate of Dystocia in Biglycan/Decorin Knockout Mice

**Figure 1:**
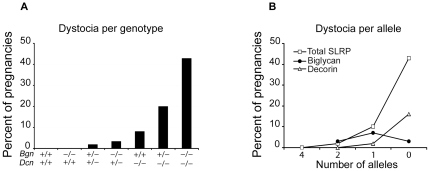
Abnormal rate of dystocia in biglycan/decorin knockout mice. **A:** Percentage of births displaying dystocia per mouse genotype. Biglycan and decorin are necessary for contractile activity leading to birth in a dose dependent manner. *Bgn−/−Dcn+/+* females are not at increased risk of dystocia, while mice lacking one or both decorin alleles are at increased risk of dystocia. *P*<0.001. Chi-square test. *Bgn+/+Dcn+/+* n = 13; *Bgn−/−Dcn+/+* n = 33; *Bgn+/+Dcn−/−* n = 11; *Bgn+/−Dcn+/−* n = 14; *Bgn+/−Dcn−/−* n = 20; *Bgn−/−Dcn+/−* n = 30; *Bgn−/−Dcn−/−* n = 7. Bgn = biglycan; Dcn = decorin. **B:** The percentage of dams displaying dystocia increases with decreasing number of maternal total SLRP (*P*<0.001) and decorin (*P*<0.001) alleles, but is independent of biglycan alleles (*P* = 0.348). Chi-square test. 4 SLRP alleles n = 13; 2 SLRP alleles n = 58; 1 SLRP allele n = 50; 0 SLRP alleles n = 7. SLRP = small leucine rich proteoglycan.

First, we evaluated the parturition characteristics of the following females compared to the wild-type (*Bgn+/+Dcn+/+*) (Figure 1): homozygous biglycan knockout and homozygous decorin knockout mice (*Bgn−/−Dcn+/+* and *Bgn+/+Dcn−/−*); the mixed double knockouts: biglycan heterozygous/decorin heterozygous knockout (*Bgn+/−Dcn+/−*), biglycan heterozygous/decorin homozygous knockout (*Bgn+/−Dcn−/−*), biglycan homozygous/decorin heterozygous knockout (*Bgn−/−Dcn+/−*); and the homozygous double knockout: (*Bgn−/−Dcn−/−*). We observed that the wild-type and the *Bgn−/−Dcn+/+* female did not display dystocia. However, the *Bgn+/−Dcn+/−*, *Bgn−/−Dcn+/−*, *Bgn+/+Dcn−/−*, *Bgn+/−Dcn−/−*, and *Bgn−/−Dcn−/−* females have a significantly increased risk of dystocia compared to the wild-type (Figure 1A) (*P*<0.001). It is well established that mice are nocturnal parturients and that the wild-type's parturition phenotype is such that onset of labor as well as birth occur during the night. Typically, one does not observe the onset of labor when observing pregnant dams in the evening, and the following morning a fully delivered new litter can be observed postpartum. Thus, dystocia was defined as the occurrence of labor that is protracted in that it is witnessed in the morning, without successful delivery of live pups by the evening of the same day. Some females with dystocia delivered their complete litter, but all pups were stillborn, while others had dilated, were in labor based on physical exam, and either incompletely delivered some pups or none at all, but were not progressing and were severely ill according to the mouse illness scale [43]. No mouse that was witnessed in labor in the morning proceeded to deliver any pups that survived the first 24 hours. Labor and cervical dilation were determined by physical exam. Labor was noted if the dam was huddled in a corner of the cage with the triad of lethargy, ruffled fur and a hunched back. Cervical dilation was defined as vaginal bleeding or dilation of the vaginal orifice beyond its normal appearance. This increase in rate of dystocia displayed an inversely linear relationship to the number of decorin and total SLRP (decorin plus biglycan) alleles per genotype but was independent of the number of biglycan alleles (Figure 1B) (*P*<0.001, *P*<0.001, *P* = 0.348, respectively).

Delayed onset of labor (defined as later than embryonic day 21) was also significantly correlated with genotype. The absence of both decorin wild-type alleles was compensated when two biglycan wild-type alleles are present, but the additional loss of one biglycan allele leads to postterm onset of labor (Table 1) (*P*<0.001). Counterintuitively, the loss of both biglycan alleles in addition to both decorin alleles did not lead to delayed onset of labor. In fact, the absence of all four alleles led to preterm birth [40]. Thus, the theoretical loss of the trigger for appropriate term labor onset was likely masked by preterm birth.

**Table 1:** *Bgn+/−Dcn−/−* dams display an increase in length of gestation (delayed onset of parturition after embryonic day 21) compared to all other genotypes.

Genotype	Delayed Labor Onset***	Total Births
*Bgn+/+Dcn+/+*	0 (0%)	13
*Bgn−/−Dcn+/+*	0 (0%)	33
*Bgn+/+Dcn−/−*	0 (0%)	11
*Bgn+/−Dcn+/−*	0 (0%)	13
*Bgn−/−Dcn+/−*	0 (0%)	20
*Bgn+/−Dcn−/−*	3 (21%)	14
*Bgn−/−Dcn−/−*	0 (0%)	7

***Chi square test: *P*<0.001.

*Bgn* = biglycan. *Dcn* = decorin.

Dystocia and delayed labor onset do not occur simultaneously, while dystocia and preterm birth do occur simultaneously in the *Bgn+/−Dcn−/−* and *Bgn−/−Dcn−/−* genotypes. Nonetheless, dystocia is most likely to occur at term (Table S1).

### Abnormal Uterine Tissue Failure in Biglycan/Decorin Knockout Mice

**Figure 2:**
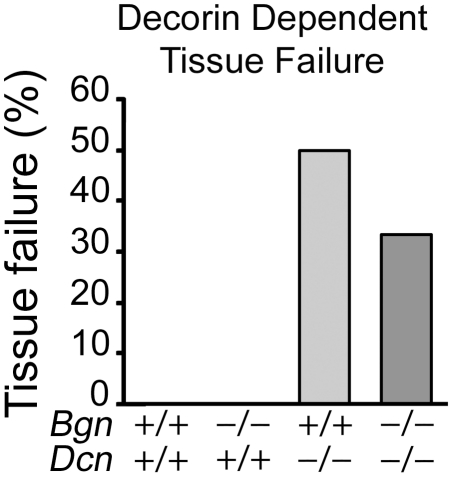
Mice lacking decorin alleles show an increased incidence of tissue failure upon loading to 10 g. *P* = 0.027. Chi-square test. *Bgn+/+Dcn+/+* n = 12; *Bgn−/−Dcn+/+* n = 17; *Bgn+/+Dcn−/−* n = 14; *Bgn−/−Dcn−/−* n = 6. *Bgn* = biglycan. *Dcn* = decorin.

Next, we sought to assess in an *in vitro* study whether an intrinsic dysfunction of the uterine muscle was leading to the observed pathological parturition. Accordingly, we tested the uterine tissue failure rate of wild-type, *Bgn−/−Dcn+/+*, *Bgn+/+Dcn−/−*, and *Bgn−/−Dcn−/−* females to a standard load of 10 g. We found that none of the wild-type uterine horn segments or *Bgn−/−Dcn+/+* uterine horn segments of either background strain (C3H or C3H/C57BL) failed (tore). Tissue failure did occur, however, in 50% and 33.3% of uterine horn segments obtained from *Bgn+/+Dcn−/−* and *Bgn−/−Dcn−/−* mice, respectively (Figure 2) (*P* = 0.027).

### Abnormal Uterine Contractions in Biglycan/Decorin Knockout Mice

**Figure 3:**
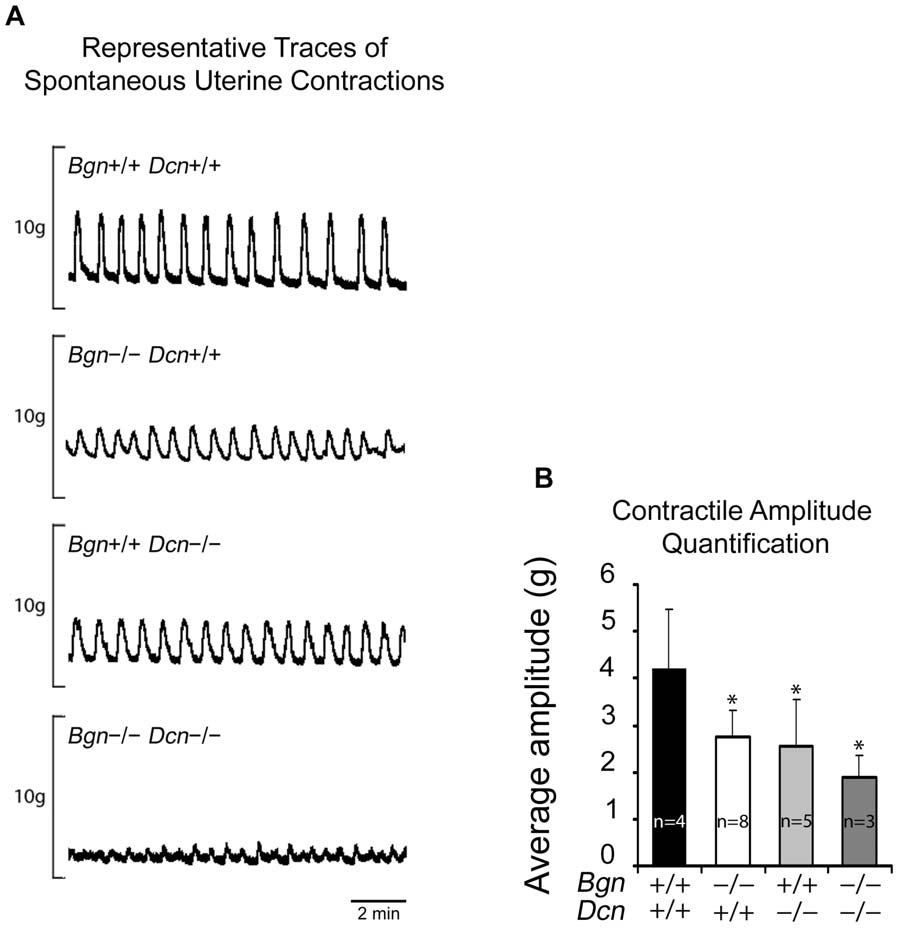
Abnormal uterine contractions in biglycan/decorin knockout mice. **A:** Representative contractile force tracings of spontaneous isometric uterine contractions display phenotypic differences per genotype. The wild-type displays regular, phasic contractions. In contrast, the absence of biglycan, decorin, or both, leads to decreased amplitude. **B:** Quantification of isometric uterine contractile amplitudes. The waveform amplitude is decreased in all knockouts compared to the wild-type. *P* = 0.012. One way ANOVA Holm-Sidak. Error bars = SD. *Bgn* = biglycan. *Dcn* = decorin.

Next, we tested whether the rate and amplitude of spontaneous non-pregnant uterine contractions differed among genotypes. The wild-type displayed regular, phasic contractions. In contrast, the *Bgn−/−Dcn+/+* uteri of both background strains (C3H or C3H/C57BL), *Bgn+/+Dcn−/−*, and *Bgn−/−Dcn−/−* displayed contractions that were lower in amplitude than the wild-type. In addition, the *Bgn−/−Dcn−/−* displayed irregular contractions (Figure 3A). Quantification of these observations revealed a significant decrease in amplitude in the *Bgn−/−Dcn+/+*, *Bgn+/+Dcn−/−*, and *Bgn−/−Dcn−/−* compared to the wild-type uteri (*P* = 0.012) (Figure 3B).

**Figure 4:**
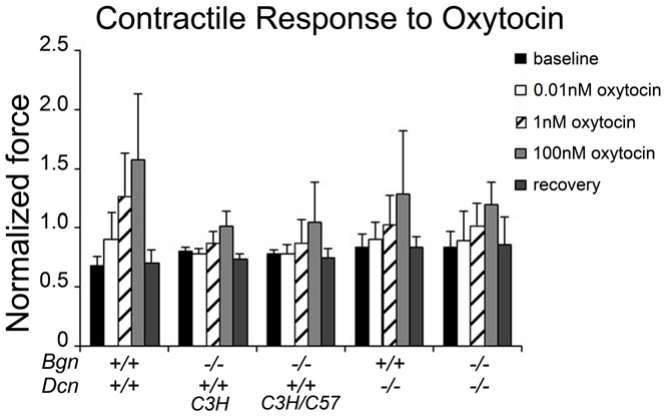
Abnormal uterine contractile force in response to oxytocin. Uterine contractile force development was examined after exposure to increasing doses of oxytocin. The wild-type displays a stepwise increase in contractile force on exposure to oxytocin. The absence of biglycan, decorin, or both results in attenuation of this response. Data is normalized to the average force of spontaneous contraction for each genotype. Baseline represents relaxation period after activation by K-PSS. *P* = 0.005. Two-way ANOVA Holm-Sidak. *Bgn+/+Dcn+/+* n = 4; *Bgn−/−Dcn+/+* C3H n = 5; *Bgn−/−Dcn+/+* C3H/C57BL n = 3; *Bgn+/+Dcn−/−* n = 5; *Bgn−/−Dcn−/−* n = 3. *Bgn* = biglycan. *Dcn* = decorin. Error bars = SD.

Next, we assessed contractile force in spontaneous as well as oxytocin induced nonpregnant uterine contractions in the following mice: *Bgn−/−Dcn+/+* C3H background, *Bgn−/−Dcn+/+* C3H/C57BL background, *Bgn+/+Dcn−/−* and *Bgn−/−Dcn−/−*. All mice were compared to wild-type mice. We observed a significant difference in contractile force between genotypes as well as between treatments (*P* = 0.005) (Figure 4). Specifically, the wild-type displayed a dose-dependent increase in normalized contractile force when exposed to 1 nM and 100 nM oxytocin compared to the force of spontaneous contractions in physiological saline solution without oxytocin (“baseline”). In contrast, the response of the *Bgn+/+Dcn−/−* was only increased on exposure to 100 nM oxytocin, but not to 1 nM, and *Bgn−/−Dcn+/+* uteri of both background strains as well as the *Bgn−/−Dcn−/−* did not increase their normalized contractile force to any dose. Also, we observed no difference between the *Bgn−/−Dcn+/+* mice of both background strains (C3H and C3H/C57BL) under any condition. Given the lack of difference in phenotype between the two strains, the two groups were merged for Figure 2 and 3B; however, they were displayed separately in Figure 4 to demonstrate the phenotypic similarity.

### Lack of a Significant Compensatory Mechanism between Biglycan and Decorin in the Knockout Mice

**Figure 5:**
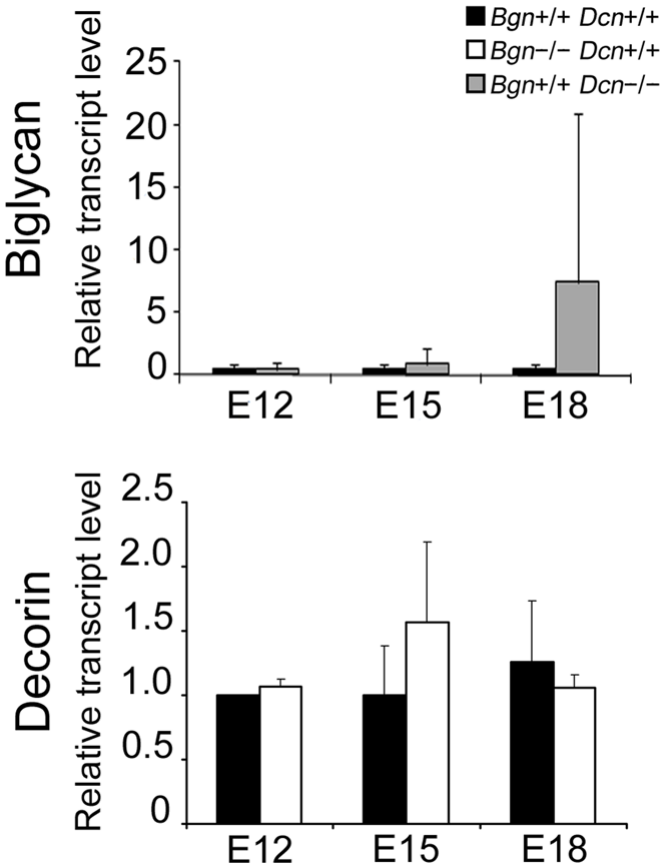
Lack of a significant compensatory mechanism at the gene level between biglycan and decorin in the knockout mice. Levels of biglycan mRNA in wild-type and *Bgn+/+Dcn−/−* mouse uterus, as well as decorin mRNA in wild-type and *Bgn−/−Dcn+/+* mouse uterus were determined using qPCR at progressive gestational ages. Biglycan and decorin are not developmentally regulated in the pregnant uterus, nor do they compensate for each other at the transcript level in the *Bgn−/−Dcn+/+* or the *Bgn+/+Dcn−/−* (*P* = 0.271 and *P* = 0.351 respectively). Two-way ANOVA Holm-Sidak. n = 4−6 samples from 4–6 pregnant dams per condition. E = embryonic day. Error bars = SD.

In order to assess possible compensatory mechanisms between biglycan and decorin, we delineated gene expression of these SLRPs in mouse uterus during the course of gestation. We measured biglycan mRNA levels in the wild-type and *Bgn+/+Dcn−/−*, and decorin mRNA levels in the wild-type and *Bgn−/−Dcn+/+*, in uteri at E12, E15 and E18 via quantitative real time PCR (qPCR). Our data indicate that biglycan and decorin gene transcriptional activity was not developmentally regulated in the uterus during the course of gestation. Furthermore, there was no significant difference in biglycan transcript levels in the presence or absence of decorin, and no difference in decorin transcript levels in the presence or absence of biglycan. This suggests that there is no compensation in the uterus between biglycan and decorin at the transcript level (Figure 5). However, a trend toward increased gene expression of biglycan in the absence of decorin at E18 was noted.

**Figure 6:**
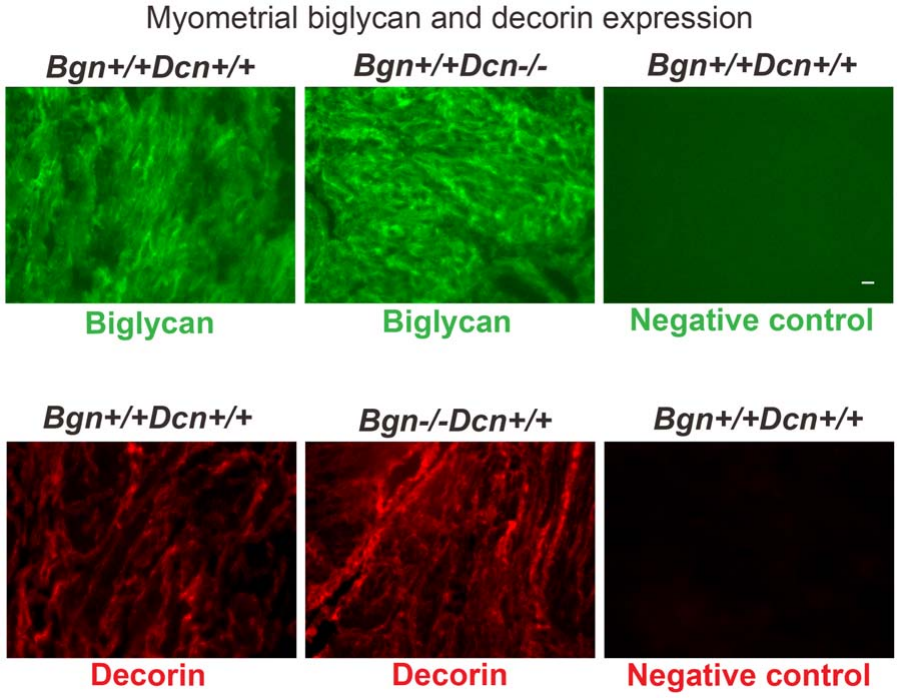
Lack of a significant compensatory mechanism at the protein level between biglycan and decorin in the knockout mice. Immunohistochemical comparison of biglycan expression in wild-type and *Bgn+/+Dcn−/−* mouse uterus as well as decorin expression in wild-type and *Bgn−/−Dcn+/+* mouse uterus at E18. There is no change in biglycan signal in the absence of decorin or decorin signal in the absence of biglycan in the uterus. 20×, scale bar 100 µm. The photomicrographs are representative of one uterus each from three pregnant females per experimental group. *Bgn* = biglycan. *Dcn* = decorin.

In order to assess protein expression in the absence of gene expression changes, we performed immunohistochemical analysis of gestational tissues at E18. We assessed decorin expression and localization in the myometrium in the presence and absence of biglycan, as well as the expression and localization of biglycan in the presence and absence of decorin. On comparison of uterine wall sections of wild-type and *Bgn−/−Dcn+/+* mice, we noted no difference in myometrial decorin expression and localization in the absence of biglycan. Conversely, biglycan displayed no difference in expression or localization in the myometrium in the absence of decorin (Figure 6). Our results were similar at E12 and E15 (data not shown). While the expression of biglycan and decorin in the wild-type is similar in both myometrium and endometrium, a slight increase in biglycan and decorin expression was noted around blood vessels (Figure S1).

### Altered TGF-β expression in biglycan and decorin knockout mice

**Figure 7:**
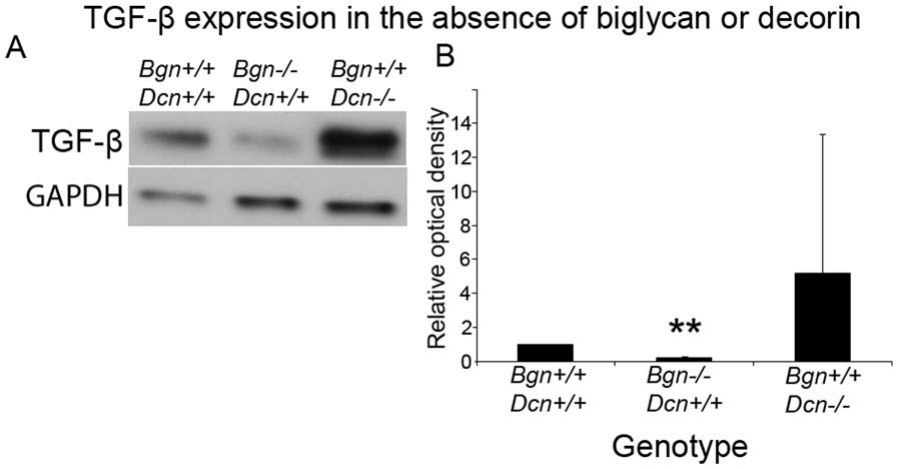
Altered TGF-β expression in biglycan knockout mice. **A:** TGF-β expression was evaluated in uterine samples of pregnant dams at E18. Western blotting was performed on wild-type, *Bgn−/−Dcn+/+* and *Bgn+/+Dcn−/−* uteri. GAPDH was used as an internal standard. **B:** TGF-β is decreased in the absence of biglycan. Digitally scanned and densitometrically analyzed Western blots from three experiments from three individual mouse uteri are expressed as a ratio of TGF-β over GAPDH and normalized to the wild-type ratio. Student's t-test. *P* = 0.004 for biglycan knockout; *P* = 0.46 for decorin knockout. Error bars = SD.

Finally, we determined TGF-β expression in the uterus at E18 to assess the role of TGF-β in uterine physiology in the absence of biglycan or decorin. TGF-β is downregulated in the *biglycan* knockout gravid uterus and unchanged in the *decorin* knockout gravid uterus, although a trend toward an increase in the *decorin* knockout was noted (Fig. 7).

## Discussion

The processes that allow for a smooth transition from uterine quiescence during pregnancy (and thus avoidance of preterm birth) to active contractions at term (and thus successful expulsion of the term fetus) are modulated by multiple factors, including neuronal, hormonal, metabolic and mechanical pathways [3]. In this paper, we present a novel model of uterine dysfunction leading to adverse gestational outcomes via dystocia and delayed labor onset.

Our findings demonstrate dystocia in mice lacking both biglycan and decorin with an inversely correlated relationship to the number of wild-type SLRP (biglycan plus decorin) alleles per genotype. These findings parallel our previously reported observation that the number of SLRP (biglycan plus decorin) alleles per genotype is inversely related to the rate of preterm birth [40]. However, in contrast to the preterm birth phenotype, in which wild-type biglycan and decorin alleles are completely complementary and interchangeable, in this dystocia phenotype the risk of dystocia is dependent on the number of decorin alleles and the total number of SLRP alleles, but independent of the number of biglycan alleles. The number of biglycan alleles only plays a role in the complete absence of decorin alleles; that is, only if both decorin alleles are absent does the rate of dystocia increase with the additional loss of each subsequent biglycan allele. Furthermore, the absence of decorin alleles and to a lesser extent the absence of biglycan alleles influences not only the quality of contractions when they occur, but also the processes that lead to the initiation of labor.

In the dams with loss of three or four SLRP alleles, we observed genotype-phenotype correlation with distinct abnormal gestational outcomes. *Bgn+/−Dcn−/−* dams display term dystocia or postterm labor onset or preterm birth associated with dystocia, while *Bgn−/−Dcn+/−* dams display preterm birth or term dystocia, but no postterm labor onset. The *Bgn−/−Dcn−/−* female displays dystocia and preterm birth individually or in combination. These observations suggest that while biglycan and decorin act in a dose-dependent compensatory manner, they nonetheless play discrete roles in the attainment of successful gestation with uterine contractility more strongly linked to decorin function than to biglycan function. At first glance, the observation that the *Bgn−/−Dcn−/−* females do not display delayed onset of labor may seem to negate the conclusion that decorin is necessary for the trigger of labor initiation. However, our observations suggest that the failure to initiate labor at term in the absence of decorin is masked by the more profoundly abnormal preterm birth gestational phenotype of the *Bgn−/−Dcn−/−*. Thus, the loss of both decorin alleles increases the risk of dystocia during labor despite the presence of both biglycan alleles. If one biglycan allele is lost in addition to both decorin alleles, the compensatory mechanisms that biglycan maintains are lost, and thus delayed onset of labor at term is seen in addition to dystocia. If the second biglycan allele is lost, also, the ensuing *Bgn−/−Dcn−/−* phenotype is so devastating that preterm birth ensues. Hence, the adverse outcome of delayed labor onset at term that is due to decorin deficiency cannot be observed because the pregnancy ends in an adverse outcome due to biglycan plus decorin deficiency prior to term. The penetrance of this mouse model is not 100%. Hence, not all mice of a specific genotype display dystocia and/or delayed labor onset. However, this is nonetheless a viable model for human dystocia and delayed labor onset given that many human genetic diseases also display a penetrance of less than 100%.

Our observations of contractile dysfunction of the pregnant biglycan- and decorin-deficient uterus *in vivo* is mirrored by the non-pregnant biglycan- and decorin-deficient uterus *in vitro*, indicating that the muscle is intrinsically affected by the absence of these SLRPs. Uterine tissue failure occurs in a decorin allele-dependent manner, while the amplitude of contractions is related to the number of total SLRP alleles, suggesting compensation between biglycan and decorin. Thus, there is an intrinsic uterine “fragility” in the uteri of double knockout animals and this fragility is primarily linked to decorin deficiency. Notably, the skin of decorin knockout mice exhibits reduced tensile strength and uniformly fails at ∼7 Newton, in contrast to the wild-type skin that fails at ∼27 Newton [44]. Similarly, oxytocin induced contractility is decreased in all genotypes compared to the wild-type, suggesting that this function is sensitive to the loss of any alleles. Also, on comparison of the two *Bgn−/−Dcn+/+* background strains (C3H and C57BL), no difference in contractility was noted, suggesting that the various backgrounds do not play a role in the uterine contractile function phenotype. The *in vitro* contractility experiments were only performed in the non-pregnant uterus given the challenges of achieving pregnancy in the various mixed genotype mice. Nonetheless, it is highly likely that our observations would be very similar in the pregnant uterus, given that our non-pregnant *in vitro* observations support our pregnant *in vivo* findings. These findings, coupled with the observation of mild (skeletal) muscular dystrophy in mice in the absence of biglycan [38], suggest that biglycan and decorin play a crucial role in uterine muscle physiology.

We did not observe an increase in biglycan gene or protein expression in the absence of decorin or an increase in decorin gene or protein expression in the absence of biglycan in the uterus during the course of gestation. This is in contrast to the compensatory increase in placental protein expression we previously reported [40]. This observation could be a result of compensatory mechanisms not being present in the uterus. Alternately, it may be a reflection of the fact that there is an abundance of both biglycan and decorin in this tissue compared to the placenta, and thus a possible compensatory increase may not be appreciable with the techniques utilized. Since no compensatory biglycan and decorin gene upregulation was observed in the uterus of *Bgn+/+Dcn−/−* and *Bgn−/−Dcn+/+* dams, possible compensation at the protein level that is not appreciable with the immunohistochemical techniques utilized may be secondary to post-transcriptional processes such as increased mRNA stabilization or decreased protein degradation and not increased gene expression. Alternately, mechanisms other than direct compensation may play a role, for example the increase or decrease of other structural or signaling proteins. Also, we observed that neither biglycan nor decorin are developmentally regulated in the uterus. Nonetheless, while compensation is likely not the predominant mechanism by which dystocia and delayed labor onset are averted in the single knockouts, there seems to be functional compensation in the uterus. For example, the observation that the *Bgn+/−Dcn−/−* females are less likely to suffer from dystocia than the *Bgn−/−Dcn−/−* may indicate some functional overlap between the two proteoglycans, given that the presence of the one biglycan allele is able to partially compensate for the dystocia phenotype predominantly caused by the absence of decorin alleles. This ability to compensate for each other in preservation of possible structural and functional properties of the uterus is similar to the compensation that has been reported in skin, bone and kidney [30], [45] between biglycan and decorin, as well as in tendon between another set of SLRPs, lumican and fibromodulin [46].

While dystocia and delayed labor onset in humans with EDS has not been reported, this may be due to underreporting, given that these clinical entities are very common and are easily amenable to treatment via labor induction and/or ultimately Cesarean section. Given the low incidence of EDS, no population studies of pregnancy outcomes have been published. Thus, even the significantly more serious, and thus reportable, complications such as atonic uterus at Cesarean section [35] and uterine rupture during labor [33], [34] have only been discussed as case reports and series of case reports. However, our data indicating that biglycan modulates TGF-β expression support the observation that uterine rupture during pregnancy also occurs in patients with aneurysm syndromes other than EDS that are caused by mutations in the TGF-β receptor [47]. Furthermore, cardiac fibroblasts lacking biglycan display increased proliferation as well as differentiation into a myofibroblast phenotype in a TGF-β-dependent manner [11]. Thus, biglycan plays a role in the regulation of uterine muscle function via a TGF-β dependent pathway that has yet to be elucidated in detail.

Here, we present a gene-dose dependent mouse model of pathological parturition secondary to dystocia and post-term labor onset caused by the genetic ablation of decorin with partial compensation by biglycan. Thus, the biglycan/decorin double knockout mouse is a novel model of a genetic pathway for dystocia and delayed labor onset, which will be a useful model to test therapeutics and potentially decrease the rate of Cesarean births secondary to dystocia.

## Supporting Information

Figure S1 **Biglycan and decorin localization in the mouse uterus.** Immunohistochemical comparison of biglycan and decorin expression in the wild-type mouse uterine wall at E18. Both biglycan and decorin are localized to the myometrium and the endometrium to a similar degree. A slight increase in biglycan and decorin signal is noted around blood vessels (yellow arrows). 4×, scale bar 500 µm. The photomicrographs are representative of one uterus each from three pregnant females per experimental group.
(TIF)

Table S1 **Correlation of timing of birth and dystocia phenotypes per genotype. Dystocia is most likely to occur at term.** Dystocia and delayed labor onset (after embryonic day 21) do not occur simultaneously, while dystocia and preterm birth do occur simultaneously in the *Bgn+/−Dcn−/−* and *Bgn−/−Dcn−/−* genotypes. *Bgn* = biglycan. *Dcn* = decorin.
(DOCX)
