# Supplementary figures and images for: Uterine Dysfunction in Biglycan and Decorin Deficient Mice Leads to Dystocia during Parturition
Source: PLoS One. 2012 Jan 13;7(1):e29627. doi: 10.1371/journal.pone.0029627 (PMC3258236; doi:10.1371/journal.pone.0029627)

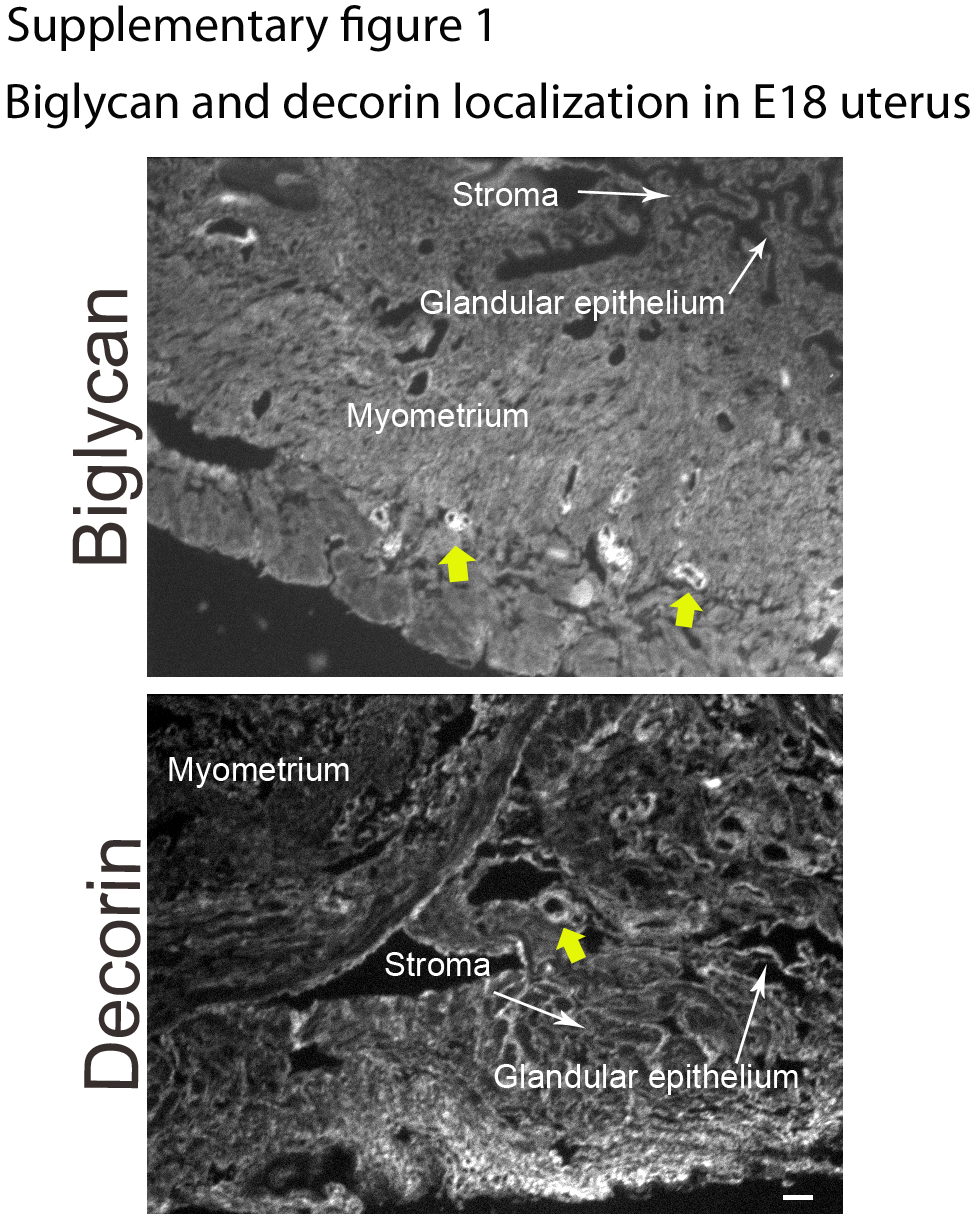

Supplement: Figure S1 — Biglycan and decorin localization in the mouse uterus. Immunohistochemical comparison of biglycan and decorin expression in the wild-type mouse uterine wall at E18. Both biglycan and decorin are localized to the myometrium and the endometrium to a similar degree. A slight increase in biglycan and decorin signal is noted around blood vessels (yellow arrows). 4×, scale bar 500 µm. The photomicrographs are representative of one uterus each from three pregnant females per experimental group. (TIF) [file pone.0029627.s001.tif]
