# Supplementary material for: Uterine Dysfunction in Biglycan and Decorin Deficient Mice Leads to Dystocia during Parturition
Source: PLoS One. 2012 Jan 13;7(1):e29627. doi: 10.1371/journal.pone.0029627 (PMC3258236; doi:10.1371/journal.pone.0029627)
Supplement: Table S1 — Correlation of timing of birth and dystocia phenotypes per genotype. Dystocia is most likely to occur at term. Dystocia and delayed labor onset (after embryonic day 21) do not occur simultaneously, while dystocia and preterm birth do occur simultaneously in the Bgn+/−Dcn−/− and Bgn−/−Dcn−/− genotypes. Bgn = biglycan. Dcn = decorin. (DOCX) [file pone.0029627.s002.docx]

**Supporting Table 1**

Correlation of timing of birth and dystocia phenotypes per genotype. Dystocia is most likely to occur at term. Dystocia and delayed labor onset (after embryonic day 21) do not occur simultaneously, while dystocia and preterm birth do occur simultaneously in the *Bgn+/-Dcn-/-* and *Bgn-/-Dcn-/-* genotypes.

| Genotype | Dystocia | Preterm birth | Delayed labor onset | Dystocia plus preterm birth | Dystocia plus delayed labor onset |  |
| --- | --- | --- | --- | --- | --- | --- |
| *Bgn+/+Dcn+/+* | 0% | 0% | 0% | 0% | 0% |  |
| *Bgn-/-Dcn+/+* | 0% | 0% | 0% | 0% | 0% |  |
| *Bgn+/+Dcn-/-* | 9% | 0% | 0% | 0% | 0% |  |
| *Bgn+/-Dcn+/-* | 8% | 0% | 0% | 0% | 0% |  |
| *Bgn-/-Dcn+/-* | 5% | 10% | 0% | 0% | 0% |  |
| *Bgn+/-Dcn-/-* | 14% | 0% | 21% | 7% | 0% |  |
| *Bgn-/-Dcn-/-* | 29% | 29% | 0% | 14% | 0% |  |

*Bgn* = biglycan. *Dcn* = decorin
